# Configuration and reduced-order modeling of a flow system based on experimental data

**DOI:** 10.1038/s41598-025-19211-3

**Published:** 2025-10-09

**Authors:** Faisal Saleem, Alicja Wiora, Józef Wiora

**Affiliations:** Department of Measurements and Control Systems, Silesian University of Technology, Gliwice, 44-100 Poland

**Keywords:** Automated measurement method, Model identification, Principal component analysis, Linear parameters varying, Model-order reduction, Engineering, Electrical and electronic engineering

## Abstract

Regulated flow systems exhibit a variable dynamic behavior when subjected to distinct inputs. The significant non-linear behavior of the system at low inputs and the difference in the output pattern for the increase and decrease in flow pose challenges in modeling. One way is to identify separate sets of parameters for each input-output data set and develop distinct models. However, this approach is computationally expensive when designing a single controller covering the whole input range. To overcome the said limitation, we used a Principal Component Analysis (PCA) based estimation technique. The developed Linear Parameter Varying (LPV) dynamic model describes real measurement values of a laboratory flow system. The data treatment consists of (1) an automated input-output data acquisition, (2) $\textrm{3}^{rd}$-order model estimation from measured data, (3) the reduction of the dynamic parameters using PCA, and (4) the development of a reduced LPV model for the entire input range. The LPV model presents its output response comparable to the experimentally taken flow rates. The proposed modeling technique can help design a single controller sufficient to achieve the desired output applicable in the whole measuring range. The effectiveness of our approach suggests its use for synthesizing an LPV controller for flow systems.

## Introduction

Determining the liquid flow is essential in many real-world processes ranging from domestic to industrial applications. Proper operation of water distribution and treatment processes, domestic heatings, condensation systems, irrigation canals, liquid poring systems, drainage systems, etc. requires accurate control of the flow system. For many applications, a specific flow rate of fluid is mandatory for steady-state operation. This is possible with an intelligent control system. Control design approaches rely on accurate models to design a controller that ensures the desired operation of the system. They depend upon a model that captures the process dynamics correctly. Consequently, an appropriate dynamic model of a process is key information for controller design^1,2^.

Literature reports many modeling approaches of flow systems providing simple to complex models representing the practical systems. These approaches applied to flow systems include the model identification with System Identification Toolbox (SIT)^3,4^, Galerkin projection^5^, diffusion wave and kinematic wave approximations for nonlinear model^6^,Linear Parameter Varying (LPV) identification of irrigation canals^7^, multi-model LPV for a tank reactor^8^, flow rate identification in pouring machine^9^, support vector machines^10^, and loss of lines with Sequential Quadratic Programming (SQP)^11^. In addition to the approaches applied to flow systems, the literature presents numerous methods for the identification of models of several real-time systems, including DC motors^12^, lithium-ion batteries^13^, twin rotors^14^, airships^15^, 3-DOF gyroscope^16^, and flexible linkage system^17^.

Model identification of the flow rate with SIT^3,4^ provides a transfer function (TF) with at least 13 poles and 9 zeros. Such an order poses high computational complexity in controller design. Galerkin projection^5^ and the diffusion wave and kinematic wave approximations^6^ solve differential and partial differential equations. Therefore, these approaches are suitable for the identification of parameters of the systems for which the prior model structure is known. LPV^7^ and multi-model LPV^8^ identification approaches develop a mathematical LPV model from a physical system, and then identify some parameters. This requires both mathematical and system identification knowledge. Moreover, the complexity of the approaches presented in^7,8^ poses a computational burden which will be reflected in an LPV control design. So, a simple identification method that provides a reduced-order model should be proposed.

This article presents a reduced-order LPV model identification approach based on simple transfer functions (TFs) obtained with SIT. It is based on real data obtained from a laboratory water flow system. We provide 10 different inputs, ranging from minimum to maximum values, to the flow system and record the corresponding outputs (flow rates). Based on these input-output (IO) data, we identify simple TFs for each case. Every TF has three poles and two zeros. We apply Principal Component Analysis (PCA) to the parameters to reduce their dimensionality to obtain two poles and one zero for each TF. Lastly, we use the interpolation method to obtain an LPV TF to represent the flow system. Statistical errors and comparison with the real-time measurements validate the accuracy of the proposed technique. One of the strengths of the proposed method is its ability to rely on simple methods of model identification. Moreover, the control design of a second-order TF has a low computational complexity compared to the high-order models. The main contributions of this work are:

1. An automated data acquisition method to measure IO data of the flow system from the zero to the maximum operating range.
2. TF model identification of the flow system for each operating frequency.
3. A reduced-order LPV model estimation technique with parametric variations depending on the real-time measurements from a flow system.

## Experimental setup

**Fig. 1:**
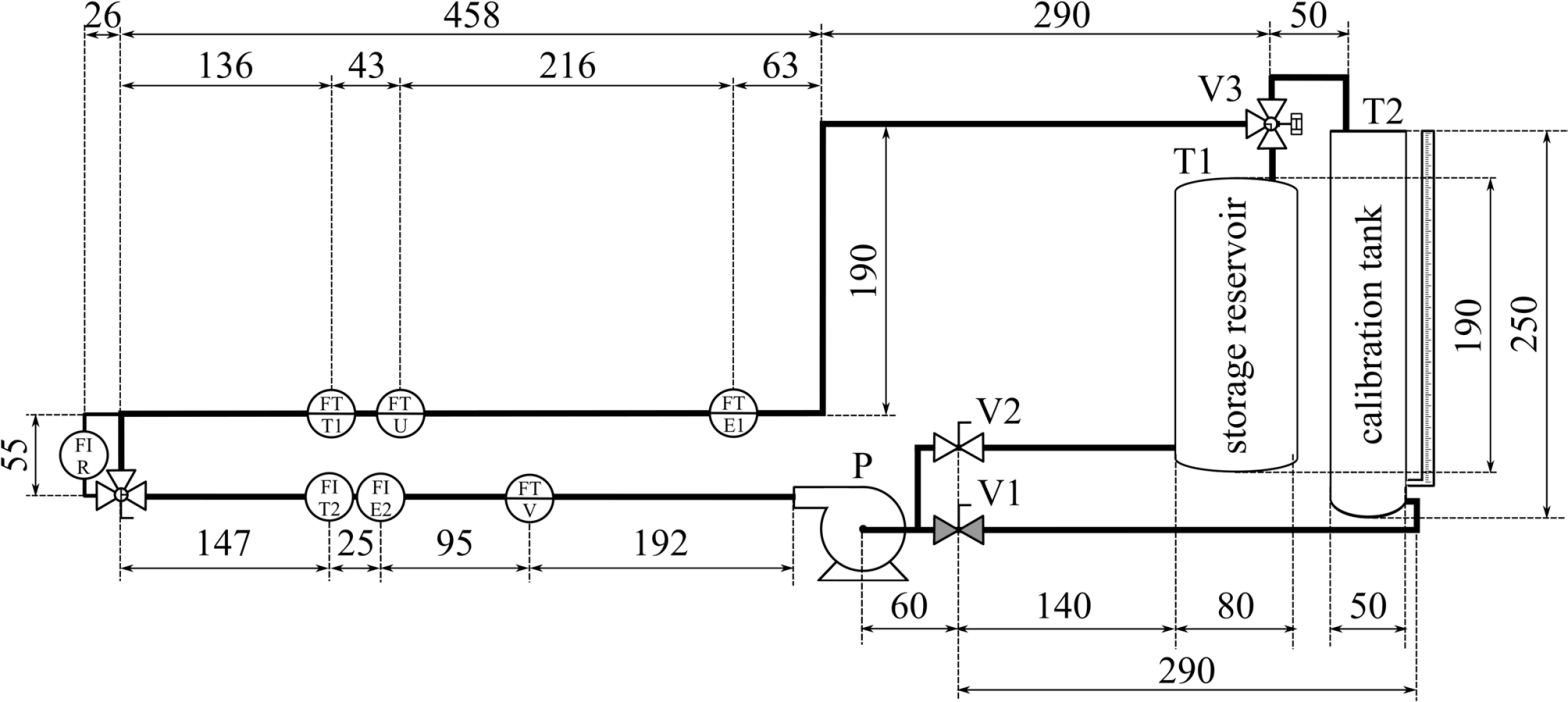
P&ID diagram of the experimental setup illustrating the water flow subsystem with dimensions (units in $\textrm{cm}$).

The tests were performed using a water flowmeter calibration system designed for didactic purposes. The flowmeters include flow transmitters and flow indicators. Piping and Instrumentation Diagram (P&ID) illustrating the experimental setup is shown in Fig. 1. The system is closed, which means it operates without an external water supply. The main loop consists of a Water pump (P), a storage reservoir (T1), a calibrated tank (T2) and flowmeters of different types. Among flow transmitters with a digital interface, the system includes the electromagnetic ENKO MPP02 (FT-E1) with the range of up to $60~\mathrm {m^3/h}$, the turbine NEGELAP NWGY (FT-T1) with a range from 1 to $20~\mathrm {m^3/h}$, the ultrasonic KHRONE OPTISONIC 3400C (FT-U) with a range of up to $18~\mathrm {m^3/h}$, and the nutating disk Badger Meter ER-500 (FT-V) with a range of up to $38~\mathrm {m^3/h}$. The nominal diameter of the pipeline (DN) is constant and equal to $40~\textrm{mm}$.

**Fig. 2:**
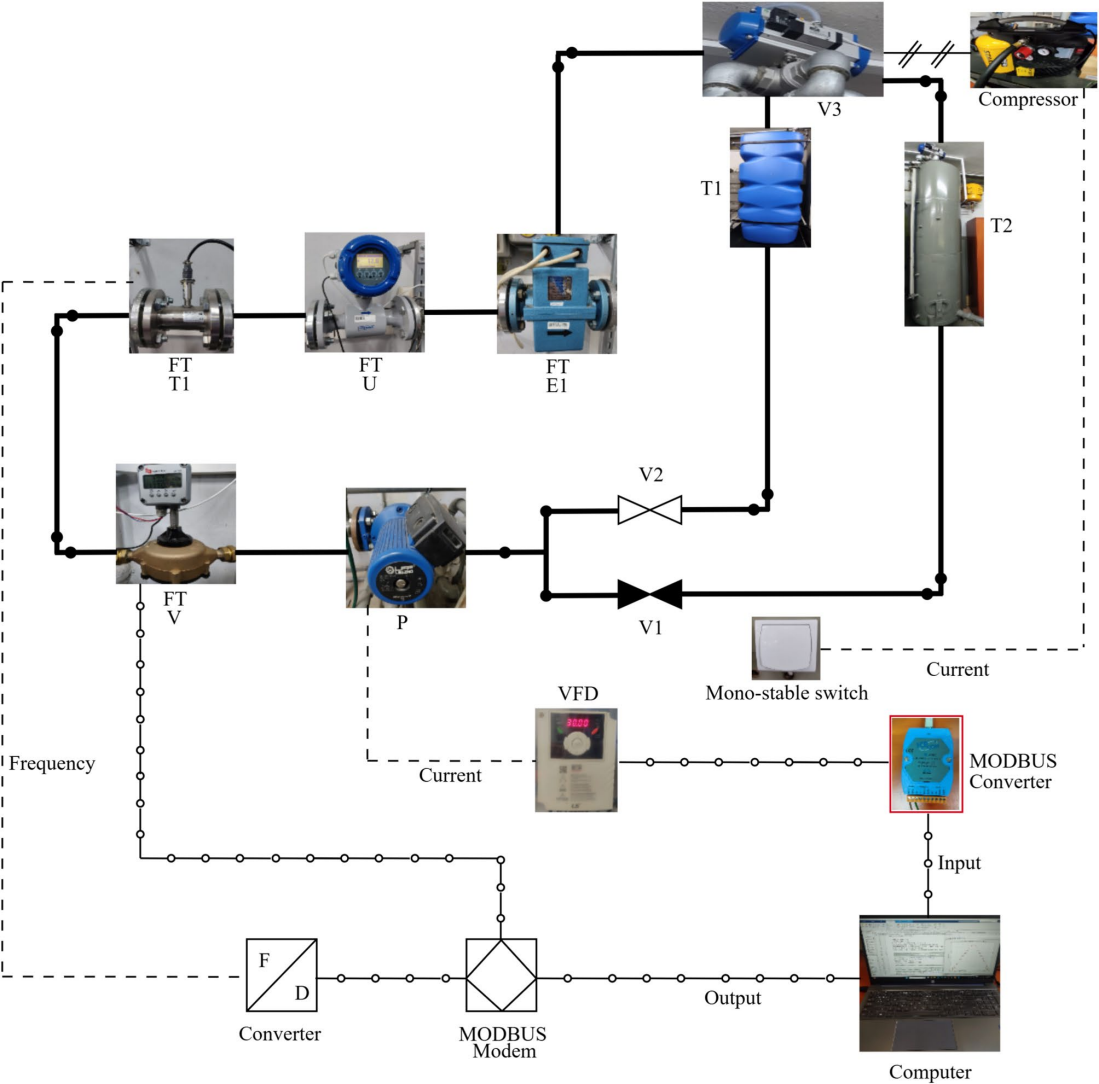
Configuration of the experimental setup for input-output data acquisition.

The installation of flowmeters on the pipeline was made in accordance with the manufacturers’ recommendations. Figure 2 illustrates the controlling principle. The value of water flow supplied by the pump is regulated by a Variable Frequency Drive (VFD). After starting the pump, water always flows in one direction from the storage reservoir (T1) through the entire installation back to the same storage reservoir. After pressing the monostable switch controlling the pneumatic valve (V3), water is directed to the measuring tank (T2) of known volume. During calibration, valve V2 is open and valve V1 is closed. Pumping water out from the calibrated tank to the storage reservoir involves changing the positions of valves V1 and V2. Since in this work we only collect flowrates of the water in the pipe, we obtain measurements in closed circuit, i.e., V1 is closed, V2 is open, and V3 is in default position (to T1). Also, we collect measurements for the flow of water from T1 to T1 excluding the monostable switch and T2 from the experimental setup.

### Configuration with MATLAB

The water flow rate, expressed in $\mathrm {m^3/h}$, passing through the pipe changes with the current frequency, expressed in $\textrm{Hz}$, powering the water pump. Therefore, the experimental setup is a single-input single-output process with frequency as the input and the flow rate as the output. To obtain the input-out data at a high sampling rate, the configuration of the VFD and the flow meter is required. We use MODBUS modem to configure FT-T1 and VFD. To allow the acquisition of the data immediately after controlling the speed of the pump, we use separate modems for FT-T1 and VDF. MATLAB software governs the configuration and data collection from the experimental setup. We used MATLAB R2024b (https://www.mathworks.com) in this work. Figure 2 shows the diagram with real photos of the setup containing the flowmeters and VFD. It shows input–output data acquisition with MODBUS communication protocol. The FT-T1 provides frequency signals proportional to flow. It is converted to digital signal by the data converter, and the data is transmitted by MODBUS protocol to the Personal Computer (PC). The system also provides collecting data from FT-V. The input data corresponding to pump frequency is transmitted from PC to VFD by a second MODBUS modem. The VFD provides a current signal to the pump with specified frequency. Listing 1 shows MATLAB code to configure VFD and FT-T1.

**Listing 1:**
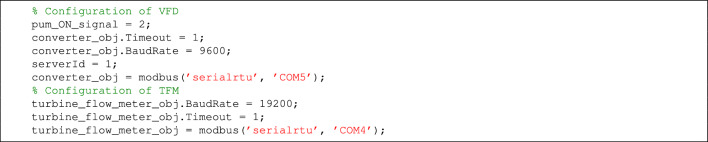
MATLAB code to configure VFD and FT-T1

### Automated data acquisition method

To create the input-output data set for the flow system, we start by initializing the software program with the variables as in Table 1.

**Table 1:** Initialization of the variables for data acquisition.

Variable	Value	Description (units)
*i*	1	Current pass
*j*	1	Current number of the fixed frequency step
$f_i$	6	Initial frequency (Hz)
*k*	60	Number of total passes
*s*	20	Total number of frequency steps
*t*	0	Initial time (s)
$t_e$	40	Total time for each frequency step (s)
$T_s$	0.016	Sampling time for data collection (s)

After the initialization, the program starts the measurement loop. The algorithm checks the current iteration. If the current iteration is less than the desired, the algorithm starts the data collection. In each iteration, we collect the flow rate for the input frequencies in the list:

1 $$\begin{aligned} f=\left[ 6,~12,~18,~24,~30,~36,~42,~48,~54,~60,~60,~54,~48,~42,~36,~30,~24,~18,~12,~6\right] \end{aligned}$$

The first half of the list in Eq. (1) represents a step-wise increase with a frequency step of 6 $\textrm{Hz}$. The frequency value of $6~\textrm{Hz}$ is equal to $\mathrm {10\%}$ of the maximum VFD frequency value. The second half includes a step-wise decrease with the same frequency step. This article uses the terms increase in flow and decrease in flow for the flow rates corresponding to the input frequencies when there is a step-wise increase in the input frequency and when there is a step-wise decrease in the input frequency, respectively. The measurement algorithm collects the input-output data for both cases: increase and decrease in flow. As long as the measurement time for one frequency step is less than the time for a particular frequency, the algorithm keeps the input frequency, acquires the flow rate for each sample, and stores both the flow rate and the sample time. A jitter is observed due to hardware properties. Once the data for one frequency step have been collected, the algorithm updates the step counter and collects data for the next frequency step.

**Fig. 3:**
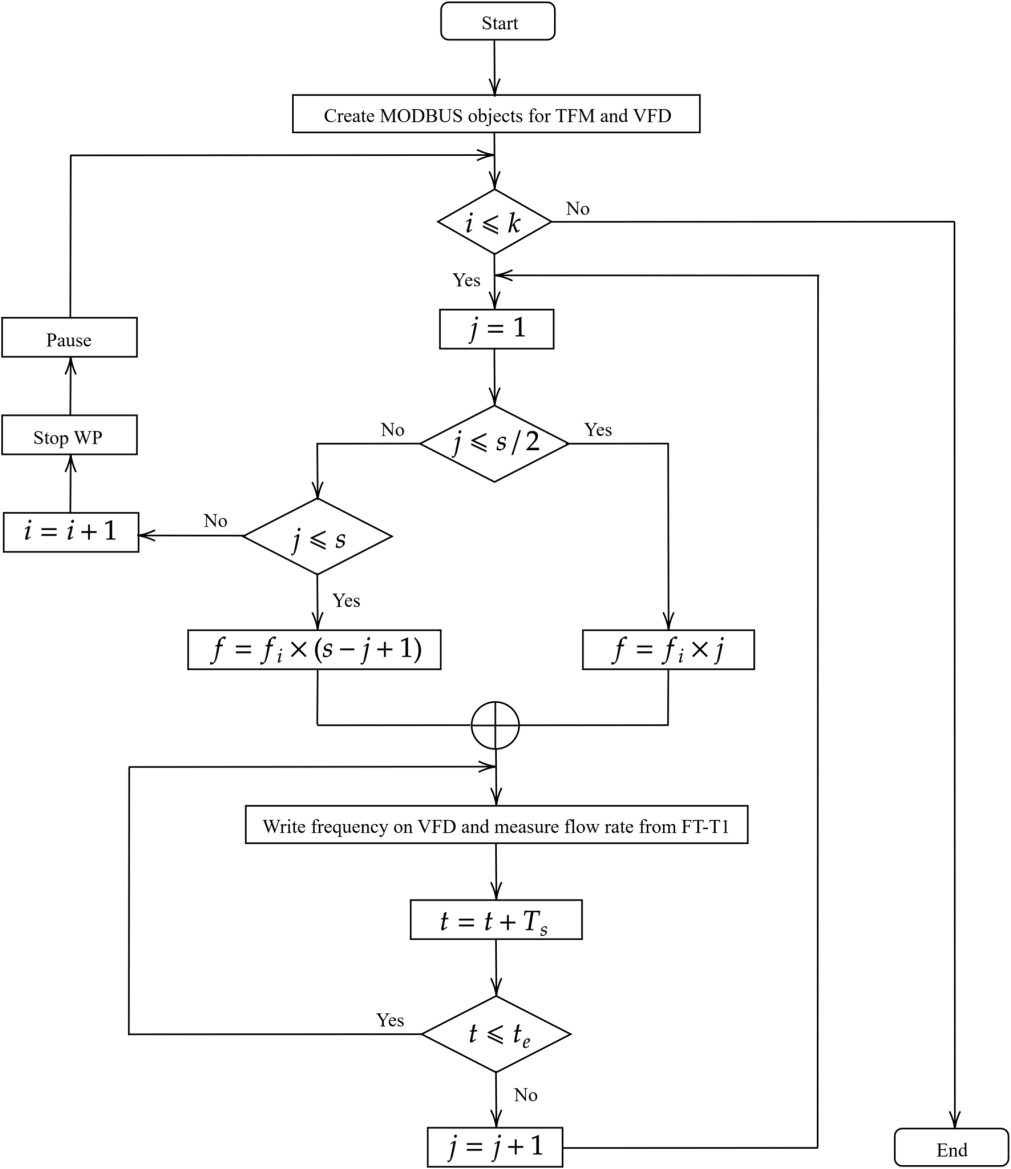
Flow chart of measurement for the input-output data acquisition. Symbols used are as in Table 1.

When flow rates for all frequency steps are recorded, the algorithm updates the iteration number and moves to the next iteration of data collection. Before collecting data for the next iteration, the algorithm stops the water pump and pauses for $30~\textrm{s}$ so that the water flow settles down to $0~\mathrm {m^3/h}$. The data collection algorithm ends when the desired iterations are achieved. Figure 3 shows the flowchart of the data collection process from the flow system. The algorithm takes $40~\textrm{s}$ for the data collection corresponding to each frequency step and $800~\textrm{s}$ for one iteration. The advantage of our measurement method lies in automated data acquisition. Once a user defines the desired number of passes, initial frequency, number of frequency steps, and the total time for each frequency step, the algorithm returns the entire data set stored in a MAT file. Lack of separate initialization and manual procedures to save the data for each iteration and frequency step makes this an automated user-friendly acquisition method.

**Fig. 4:**
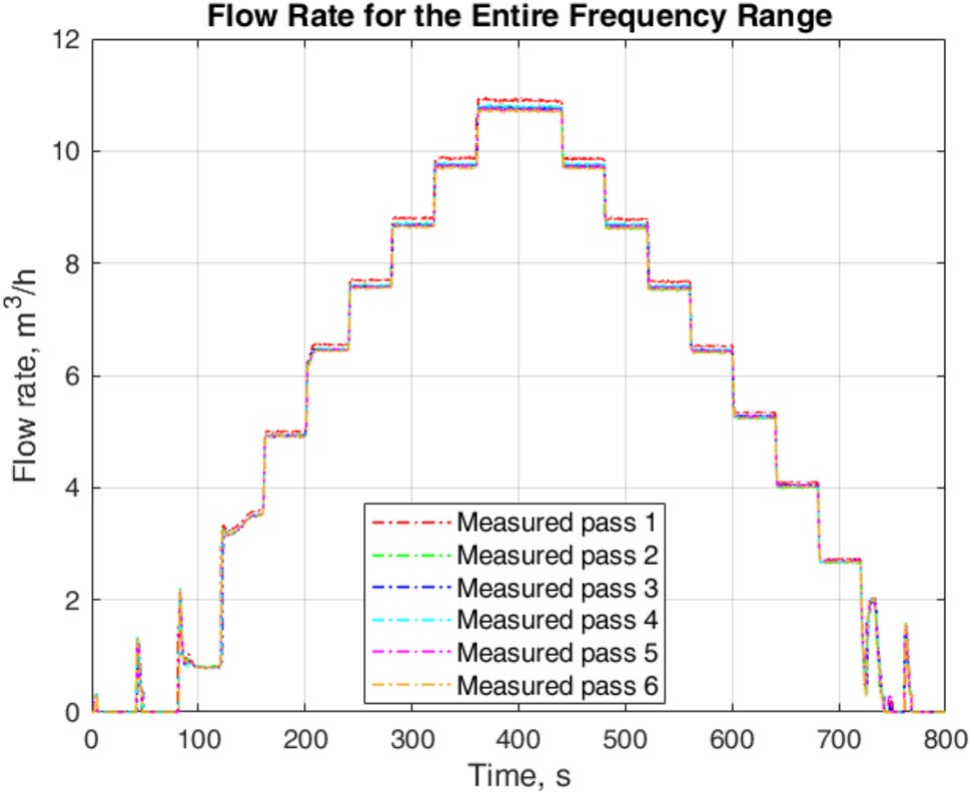
Flow rate for change in frequency for the increase and decrease of the flow.

For the model identification using the proposed approach, we collected data for 60 iterations. In this article, an iteration is the process of collecting all measurements for the input frequencies in Eq. (1), and a pass represents all values of water flow rate corresponding to an iteration. To avoid the complexity in the graphical representation, this article shows 6 passes. Figure 4 depicts the flow rate when input frequency changes from 0 to $60~\textrm{Hz}$ (for increase in flow) and 60–$0~\textrm{Hz}$ (for decrease in flow). It represents a highly nonlinear behavior of the system from 40 to $160~\textrm{s}$ for the increase in flow. These are the cases when the input frequency is $6~\textrm{Hz}$, $12~\textrm{Hz}$, and $18~\textrm{Hz}$. There is also a highly nonlinear behavior of the flow system for the decrease in flow when the input frequency is $18~\textrm{Hz}$, $12~\textrm{Hz}$, and $6~\textrm{Hz}$.

**Fig. 5:**
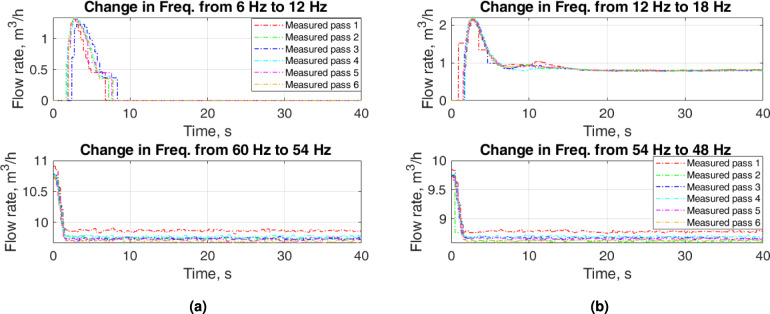
Zoomed parts of Fig. 4: upper plots—increase in flow for low input frequencies and lower upper plots—decrease in flow for high input frequencies.

Figure 5 shows some zoomed parts of Fig. 4 for the increase in flow when input frequencies are low and for the decrease in flow when input frequencies are high. When the input frequency changes from 6 to $12~\textrm{Hz}$, it takes about $2~\textrm{s}$ to start the flow that the FT-T1 detects. An abrupt flow rate of $1.4~\mathrm {m^3/h}$ appears which slowly reaches $0~\mathrm {m^3/h}$. When we change the input frequency from 12 to $18~\textrm{Hz}$ in the case of the increase in flow, an overshoot of $2.2~\mathrm {m^3/h}$ is detected as a transient flow rate that settles to $0.8~\mathrm {m^3/h}$ after $17~\textrm{s}$. This illustrates the nonlinear behavior of the flow system for the low input frequencies. Decreasing flow for high input frequencies stabilizes to a steady-state flow rate after $2~\textrm{s}$. The difference in the collected values for separate passes is due to the uncertainty in the results of the measurements.

The uncertainty assessment is commonly categorized into Type A and Type B methods of evaluation. Type A evaluation uses repeated measurements where the uncertainty component is obtained from the dispersion of measured values using standard deviation. Type B method uses other information, such as those provided by the manufacturers of the sensors, calibration reports, and other methods of scientific judgments for uncertainty evaluation. Since our work uses series of repeated measurements, the uncertainty component in this case can be obtained from Type A evaluation^18^. However, this paper treats results of the measurements as they are and identifies the parameters of the dynamic models for each pass. It then uses the average values of the parameters to report the results of the proposed approach.

The interference in the low-frequency measurements is partly due to the unstabilized stream profile (transitional flow). Correct measurements of the water flow rate should be expected for a stabilized profile. In our case, this is turbulent flow. Assuming Reynolds number $Re=4000$, for which we can expect correct water flow rates, we obtain a minimum volumetric flow rate of $0.5~\mathrm {m^3/h}$ at a temperature of $20^o\textrm{C}$. Taking into account the variable roughness of the pipes, numerous obstacles inside the pipeline, changes in the stream direction and sensor limitations, the safe value of the Reynolds number is about $10\,000$, which gives a minimum flow rate of $1~\mathrm {m^3/h}$. Since flow rates below $1~\mathrm {m^3/h}$ fall outside the measuring range of the turbine flowmeter, the accuracy of these measurements is unknown. For the measurements outside the measuring range of FT-T1, we only show the flow rates as a representation of the dynamic behavior of the system. Therefore, the dynamic models developed for these cases may not represent the dynamics of the actual system.

**Fig. 6:**
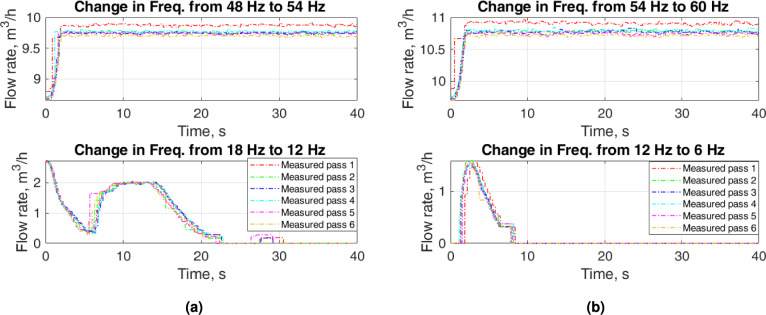
Zoomed parts of Fig. 4: upper plots—increase in flow for high input frequencies and lower upper plots—decrease in flow for low input frequencies.

Figure 6 depicts some zoomed parts of Fig. 4 for the increase in flow when input frequencies are high and for the decrease in flow when input frequencies are low. For low input frequencies the flow rate stabilizes to a steady-state value after $2~\textrm{s}$. The decreasing flow for low frequencies presents a nonlinear behavior. The steady-state flow rate for the decrease in flow is $0~\mathrm {m^3/h}$ for both input frequencies, $12~\textrm{Hz}$ and $6~\textrm{Hz}$. However, the transients in the flow rate show a complicated response when the input frequency changes from $18~\textrm{Hz}$ to $12~\textrm{Hz}$.

## Identification of reduced-ordered model

Dissimilar flow rates for different input frequencies of the flow system pose challenges in obtaining a single dynamic model for the entire input range. Therefore, this paper identifies separate dynamic models for each input frequency and the corresponding flow rate. We identify the dynamic model of the system for each frequency in Eq. (1) for every pass. Since there are 60 passes in total, we have 60 dynamic models for each input-output data. We then obtain one dynamic model for each distinct input-output combination developed by calculating the mean values of the parameters. In this way, there are 20 models for the entire input range. These models have 2 zeros and 3 poles.

### Model identification

This paper solves an unconstrained optimization problem to identify the dynamic model based on time series input-output data of the flow system. The optimization problem

2 $$\begin{aligned} \begin{aligned} \min _{n_1, n_2, n_3, d_1, d_2, d_3} \quad&\frac{1}{N}\sum _{i=1}^{N}\vert {y_a - y_e}\vert \ \end{aligned} \end{aligned}$$

where $n_1, n_2, n_3, d_1, d_2$, and $d_3$ are the parameters of the dynamic model to be identified, $y_a$ denotes the actual flow rate, $y_e$ represents the estimated flow rate, and *N* denotes the total number of samples of the flow rate, minimizes the mean absolute error between experimental and estimated responses of the flow system using SQP^19^ offered in the optimization toolbox in MATLAB^20^. The literature presents several types of errors as quality factors to minimize such as $L_1$-norm, $L_\infty$-norm, integral absolute error, integral square error, etc.^21^. Although it is less sensitive to measurement values and other gross errors, we use mean absolute error because of the simplicity of implementation. Since this research mainly focuses on the estimation of the reduced-model, we utilized the simple tools available to solve an optimization problem to identify $3^{rd}$-order models:

3 $$\begin{aligned} G(s)=\frac{n_1 s^2+n_2 s+n_3}{s^3+d_1 s^2+d_2 s+d_3}. \end{aligned}$$

This research also identifies $2^{nd}$-order dynamic models as

4 $$\begin{aligned} G_{2p}(s)=\frac{n_a s+n_b}{s^2+d_a s + d_b}. \end{aligned}$$

To obtain $G_{2p}(s)$, we solve Eq. (2) with $y_e=y_{e,2p}$ where $y_{e,2p}$ is the flow rate of the $2^{nd}$-order model in Eq. (4).

### PCA for model order reduction

Principal Component Analysis (PCA) determines a pseudo set of inputs that are orthogonal to each other and are linear combinations of the actual inputs to capture a maximum amount of system dynamics using lesser number of variables^22^. Thus, the idea of PCA is to develop a reduced dimensional pseudo-variable orthogonal input space to facilitate reduced-order modeling. Consider reducing dimensionality of a matrix $X \in \mathbb {R}^{n\times N}$ to $\hat{X} \in \mathbb {R}^{m\times N}$. The PCA extracts the important components from the data by applying the transformation based on Singular Value Decomposition (SVD) of

5 $$\begin{aligned} P=\begin{bmatrix} p_1&p_2&p_3 \end{bmatrix}^T\quad \in \mathbb {R}^{3\times N}. \end{aligned}$$

where *P* is the matrix containing *n* parameters for *N* measurements, PCA can reduce the number of parameters of the dynamic model of the system to *m*. PCA applies SVD to obtain

6 $$\begin{aligned} P=U\times S\times V^T \end{aligned}$$

where $S\in \mathbb {R}^{n\times N}$ and $U\in \mathbb {R}^{n\times n}$ represent the matrix containing singular values and singular vectors, respectively. While $V\in \mathbb {R}^{N\times N}$ represents the projection matrix of the observations of measurements such that $V\times V^T = I$ where $I\in \mathbb {R}^{N\times N}$ is an identity matrix^23^. PCA can result in the reduced dimensionality as

7 $$\begin{aligned} \hat{P}=U_{m\times n} \times P_{n\times N}\quad \in \mathbb {R}^{m\times N}. \end{aligned}$$

The number of the reduced parameters *m* is selected based on the fraction of the total variance ($v_m$). This $v_m$ decides the closeness of the information presented with the reduced number of data parameters compared to that with total parameters. It can be calculated as^24^

8 $$\begin{aligned} v_m = \frac{\sum _{i=1}^{m} s_i}{\sum _{i=1}^{n} s_i} \end{aligned}$$

where $s_i$ denotes $i^{th}$ singular value in *S*. The original parameters can be regenerated from the reduced parameters as

9 $$\begin{aligned} \tilde{P}=U^T_{m\times n}\times \hat{P}. \end{aligned}$$

We can regenerate parameters to obtain $3^{rd}$-order using Eq. (9) to find $$\tilde{P}=\begin{bmatrix} \tilde{p}_1&\tilde{p}_2&\tilde{p}_3 \end{bmatrix}^T$$. The proposed research uses PCA to reduce the number of parameters of the dynamic models to have 1 zero and 2 poles. The reduced-order models have the form:

10 $$\begin{aligned} \hat{G}(s)=\frac{\hat{n}_1 s+\hat{n}_2}{s^2+\hat{d}_1 s +\hat{d}_2 }. \end{aligned}$$

Dynamic parameters $\hat{n}_1, \hat{n}_2, \hat{d}_1$, and $\hat{d}_2$ of the reduced-order model $\hat{G}(s)$ in Eq. (10) are different than those in Eq. (3). These parameters were obtained after the application of the transformation with PCA.

### Reduced-order LPV model

Excitation of the flow system under different input frequencies listed in Eq. (1) results in distinct dynamic behavior. Model identification results in distinct dynamic models. A set of 20 input-output combinations results in 20 dynamic models. The reduction of the PCA-based model results in a smaller order of the model with the parameters as in Eq. (7). However, these parameters have distinct values corresponding to each input-output data for increase and decrease in the flow. One approach is to use each set of parameters for a particular input-output data as an individual dynamic model. We propose another approach where it is possible to consider the variation of each dynamic parameter with respect to input frequency as a single varying parameter $\theta$. The variation of each parameter depends on real-time measurements from the system. This approach treats the PCA-based reduced dynamic model as

11 $$\begin{aligned} G(s,\theta )=\frac{\hat{n}_1(\theta ) s + \hat{n}_2(\theta )}{s^2 + \hat{d}_1(\theta ) s + \hat{d}_2(\theta )} \end{aligned}$$

The proposed approach constructs a comprehensive $2^{nd}$-order model with the dynamic parameters varying with the change in the input frequency. The dynamic parameters in runtime at a particular sample time are calculated by linear interpolation as^7^

12 $$\begin{aligned} p^i(\theta _k)=\alpha _o^i + \sum _{k=1}^{k=M} \alpha _k^i \theta _k^i \end{aligned}$$

where $i=1,..., 4$ denotes the $i^{th}$ parameter, $p=[p^1, p^2, p^3, p^4]=[\hat{n}_1, \hat{n}_2, \hat{d}_1, \hat{d}_2]$, $M=20$ is the total number of dynamic transfer functions, and *k* denotes the current sampling instant in runtime. For an LPV model the following should be satisfied:

13 $$\begin{aligned} \sum _{k=1}^{k=M} \alpha _k^i = 1. \end{aligned}$$

An estimated reduced-order LPV model of the flow system in online configuration at any sampling instant becomes

14 $$\begin{aligned} G_k(s,\theta _k)=\frac{p^1(\theta _k) s +p^2(\theta _k)}{s^2 + p^3(\theta _k) s + p^4(\theta _k)}. \end{aligned}$$

The LPV model in Eq. (14) includes the contribution variation of dynamic parameters with a change in the input frequency.

## Results and discussion

Solving Eq. (2) for a $3^{rd}$-order model results in distinct dynamic models for each input-output data, as in Fig. 4, which results in a distinct set of parameters. We solved the optimization for 60 input-output data sets and calculated the average value of each parameter. This research also identifies $2^{nd}$-order dynamic models for comparison with those obtained through the proposed approach. Table 2 presents the average values of the parameters for $2^{nd}$ and $3^{rd}$-order identified models. We rounded them for two significant figures to increase readability.

These parameters are distinct when the system is excited by different frequencies for both increase and decrease in the flow. We also observe that the dynamic parameters for the same frequency are different in case of an increase in flow from those for a decrease in flow. However, collecting the input-output data at each sampling time ($T_s$) provides dynamic behavior based on real-time measurements. Therefore, we have different dynamic parameters of the flow system for increases and decreases in flow with the same input frequencies.

**Table 2:** Dynamic parameters of the flow system models obtained after solving Eq. (2).

*f*	$3^{rd}$-order model						$2^{nd}$-order model			
	$n_1$	$n_2$	$n_3$	$d_1$	$d_2$	$d_3$	$n_a$	$n_b$	$d_a$	$d_b$
Increase in flow										
6	$-0.073$	0.16	0.011	0.63	8.2	0.95	$-0.043$	0.0012	0.39	0.25
12	$-0.14$	0.15	0.013	1.6	1.5	0.42	0.069	0.0013	0.52	0.22
18	$-0.11$	0.15	$-0.021$	1.2	1.8	0.47	0.048	0.022	0.62	0.47
24	0.047	$-0.26$	0.71	2.3	8.1	6.6	$-0.13$	0.23	2.4	2.1
30	0.026	$-0.11$	0.28	3.4	7.2	5.9	$-0.043$	0.083	1.9	1.8
36	$-0.031$	$-0.038$	0.14	1.9	6.2	3.4	$-0.071$	0.087	3.3	2.1
42	0.027	$-0.11$	0.27	3.9	10	10	$-0.033$	0.062	2.1	2.3
48	0.031	$-0.11$	0.29	3.1	11	13	$-0.027$	0.059	2.2	2.6
54	0.031	$-0.14$	0.45	4.0	17	22	$-0.032$	0.074	2.8	3.7
60	$-0.0013$	$-0.038$	0.28	3.9	16	17	$-0.036$	0.12	4.7	6.9
Decrease in flow										
6	$-0.42$	0.68	$-0.0012$	2.4	2.4	0.70	0.23	0.0012	0.66	0.25
12	$-0.10$	0.0013	$-0.0032$	0.32	0.13	0.010	0.011	$-0.081$	0.58	0.49
18	$-0.024$	0.28	$-1.7$	7.3	21	22	0.075	$-0.27$	3.1	3.6
24	$-0.022$	0.16	$-0.70$	4.9	13	14	0.057	$-0.16$	2.7	3.1
30	$-0.051$	0.021	$-1.3$	5.1	22	33	0.044	$-0.17$	3.0	4.4
36	$-0.011$	0.095	$-0.59$	5.3	16	19	0.038	$-0.13$	3.1	4.2
42	$-0.010$	0.089	$-0.57$	5.6	18	22	0.034	$-0.12$	3.2	4.7
48	0.0071	0.029	$-0.69$	6.0	24	31	0.11	$-0.22$	4.6	9.5
54	$-0.0082$	0.069	$-0.48$	5.8	19	25	0.026	$-0.098$	3.4	5.2
60	$-0.0013$	0.0012	$-0.0042$	3.3	21	20	0.0013	$-0.0014$	2.2	6.5

With 60 passes, we have 60 sets of parameters with average values as in Table 2. The proposed approach applies PCA on the matrices created by the numerator and denominator parameters separately. We create $N_j=[n_{i,1},n_{i,2},n_{i,3}]^T \in \mathbb {R}^{3\times N}$, where $i=1,...,20$ denotes the total number of frequency steps. We then reduce the dimension of $N_j$ to $\hat{N}_j \in \mathbb {R}^{2\times N}$ using Eq. Eq. (7). This results in 60 sets of reduced numerator parameters. We apply the same method to reduce the dimensionality of the matrix containing denominator parameters. The average values of the 60 sets of the parameters of the reduced dynamic models for each input frequency are given in Table 3. Selecting $m=2$ for PCA-based model reduction in Eq. (7) results in $v_m\ge 0.92$ (from Eq. (8)) for each reduction.

The dynamic parameters of the PCA-based reduced-order model in Table 3 differ from those of the $2^{nd}$-order identified model in Table 2 for the same frequencies. Dynamic parameters of the reduced model for the same frequencies are also distinct from any of the parameters of the $3^{rd}$-order identified model in Table 2. This is due to the transformation applied during dimensionality reduction with PCA using Eq. (7). However, the reduced-order model produces a comparable flow rate for $f\ge 12~\textrm{Hz}$ to that of the $3^{rd}$-order identified model for the same input frequencies (see Fig. 9).

**Table 3:** Parameters of the PCA-based reduced order models obtained by Eq. (7).

*f*	$\hat{n}_1$	$\hat{n}_2$	$\hat{d}_1$	$\hat{d}_2$	$\hat{n}_1$	$\hat{n}_2$	$\hat{d}_1$	$\hat{d}_2$
	Increase in flow				Decrease in flow			
6	0.16	0.063	9.1	0.49	$-0.056$	0.059	0.14	$-0.14$
12	0.0012	0.0081	$-0.18$	0.039	0.0010	$-0.0010$	0.081	0.043
18	$-0.16$	0.016	0.45	0.20	$-0.42$	$-0.60$	$-16$	2.5
24	0.48	0.020	$-10$	$-4.0$	0.0051	$-0.0051$	$-0.24$	$-0.075$
30	0.014	$-0.0052$	0.35	$-0.035$	$-0.36$	0.70	$-15$	$-26$
36	0.072	$-0.032$	$-2.6$	$-2.9$	$-0.022$	$-0.028$	1.8	1.1
42	0.027	0.0061	0.34	0.12	0.022	0.025	2.45	$-0.59$
48	0.036	0.0084	$-0.78$	0.18	$-0.25$	0.031	$-11$	25
54	0.18	0.050	5.0	$-24$	$-0.058$	0.020	$-6.6$	$-1.4$
60	$-0.14$	$-0.32$	$-28$	4.0	0.0014	0.0012	$-22$	2.0

**Fig. 7:**
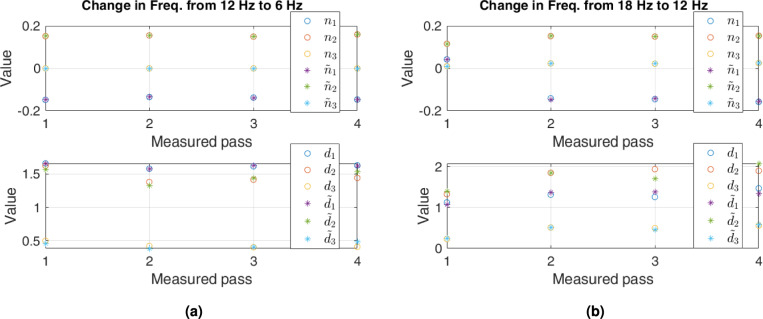
Comparison of original parameters (circles) with regenerated parameters (stars) corresponding to different frequencies. Upper plots are for the numerators, while lower—for the denominators.

**Fig. 8:**
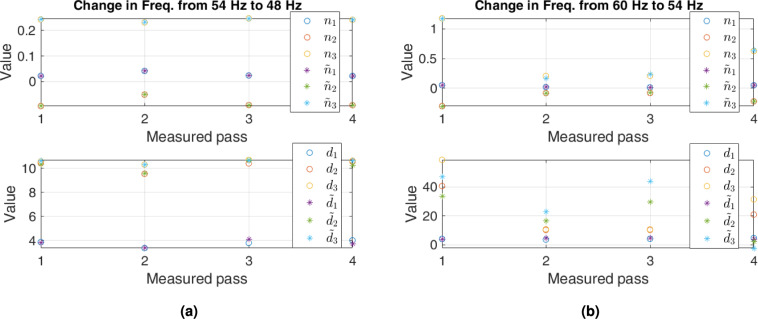
Comparison of original and regenerated parameters as Fig. 7, when the input frequency is $48~\textrm{Hz}$ and $54~\textrm{Hz}.$.

PCA can regenerate dynamic parameters comparable to the original parameters by applying reverse transformation Eq. (9). Figures 7 and 8 represent the original and regenerated parameters for some cases of decrease in flow. For a clear presentation, we show the parameters for the first four passes. Figure 7 shows that PCA regenerates numerator parameters similar to the originals for all passes when the frequency changes from 12 to $6~\textrm{Hz}$. The regenerated parameters of the denominator for the same frequency are close to the original values for the $3^{rd}$ pass, while they are somewhat comparable for the other three passes. The same is the case for the numerator when the frequency changes from 18 to $12~\textrm{Hz}$. However, the regenerated denominator parameters differ from the original for all passes. The difference is apparent for the $3^{rd}$ and $4^{th}$ pass.

Figure 8 shows that the regenerated numerator parameters are similar to the original when frequency changes from 54 to $48~\textrm{Hz}$ and from 60 to $54~\textrm{Hz}$. For the denominator, the regenerated parameters have an observable difference for $3^{rd}$ and $4^{th}$ passes when frequency changes from 54 to $48~\textrm{Hz}$. However, this difference is clear for all passes when frequency changes from 60 to $54~\textrm{Hz}$.

**Fig. 9:**
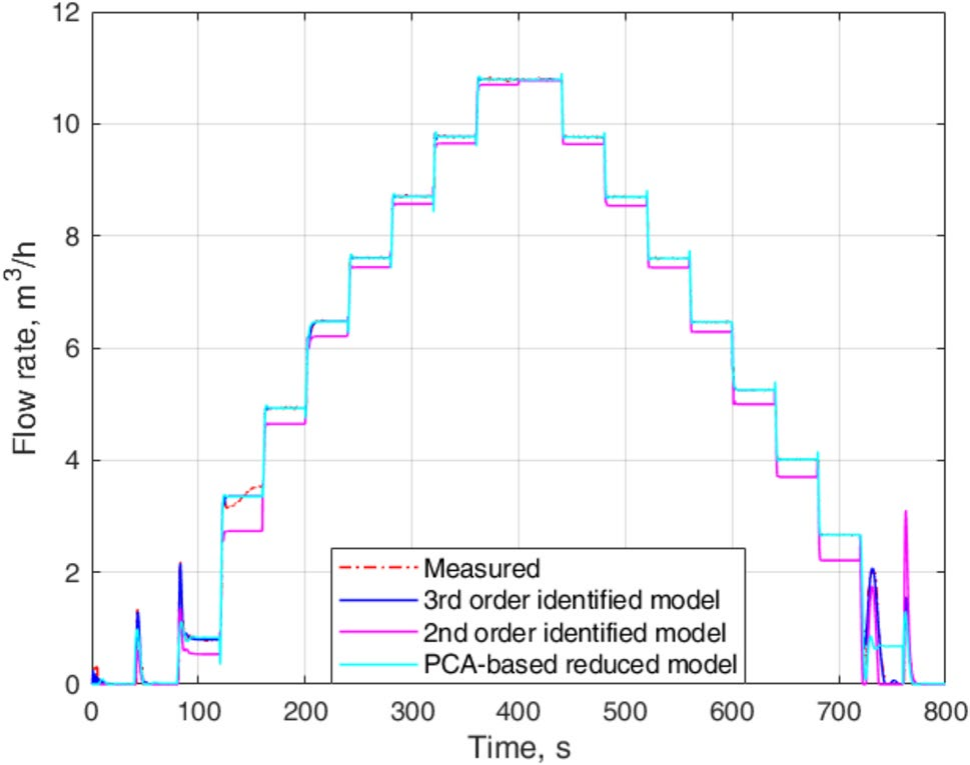
Comparison of PCA-based reduced model with measured flow rates, $3^{rd}$-order, and $2^{nd}$-order reduced models.

Figure 9 presents a graphical comparison of the proposed approach with experimental measurements, and flow rates of $2^{nd}$-order and $3^rd$-order identified models. While the $3^{rd}$-order identified model provides the flow rate closer to the experimental measurements, it has one additional pole and zero compared to that of the PCA-based reduced LPV model.

The dynamic model obtained with our method reaches the steady-state value of the flow rate same as the experimental measurements. Although it presents some transient errors for the increase in flow in case of low input frequencies, the response outperforms $2^{nd}$-order identified model. In contrast to the $2^{nd}$-order identified model, the PCA-based reduced LPV model only presents steady-state error for the decrease in flow for $f=6~\textrm{Hz}$.

**Fig. 10:**
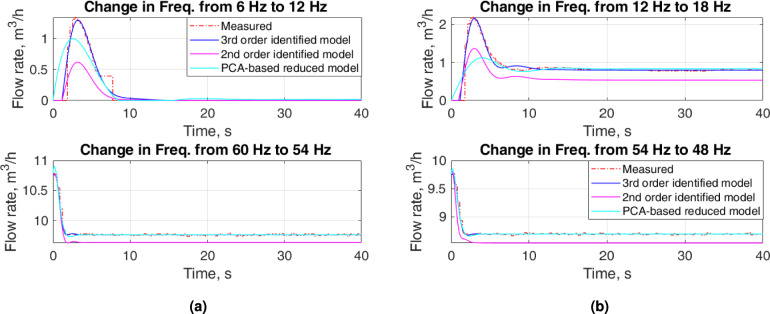
Zoomed parts of Fig. 9: upper plots—increase in flow for low input frequencies and lower upper plots—decrease in flow for high input frequencies.

**Fig. 11:**
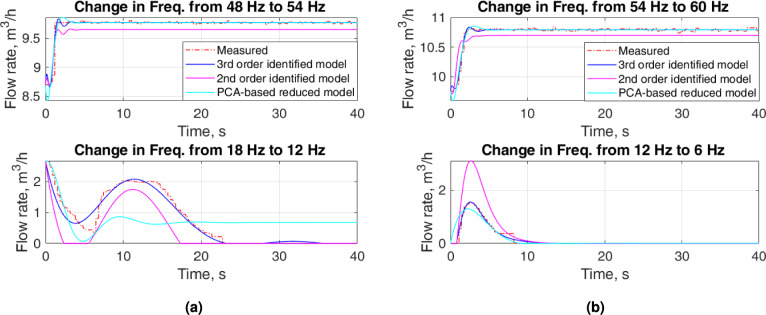
Zoomed parts of Fig. 9: upper plots—increase in flow for high input frequencies and lower upper plots—decrease in flow for low input frequencies.

The proposed approach resulting in a $2^{nd}$-order LPV model outperforms the $2^{nd}$-order identified model. The proposed approach provides a comparable flow rate to that for the $3^{rd}$-order identified model for $f\ge 12~\textrm{Hz}$. However, it presents a considerable amount of overshoots and undershoots for some cases. Figures 10 and 11 show a comparison of the flow rates of proposed approach with the experimental measurements and flow rates of identified models for specific frequencies. Because the focus of this research is to develop a dynamic model that will be used to design a suitable controller for the flow system, Figures 10 and 11 advocate the selection of a reduced model based on PCA to reduce the complexity of the implementation. Although the $2^{nd}$-order model is easy to identify and implement compared to the proposed approach, the lack of representation of the dynamics of the experimental setup opposes its use for control design.

Table 4 shows the mean absolute errors between the actual and estimated flow rates as

15 $$\begin{aligned} E_{e}=\frac{1}{N}\sum _{i=1}^{N}\vert { y_a - y_{e}}\vert \end{aligned}$$

where $y_e$ and $E_e$ denote the flow rate and mean absolute error of the $3^{rd}$-order identified model. The mean absolute error for the $2^{nd}$-order identified and PCA-based reduced order LPV models is $E_{e,2p}=E_e$ when $y_e=y_{e,2p}$ and $E_{e,pca}=E_e$ when $y_e=y_{e,pca}$ where, $y_{e,2p}$ and $y_{e,pca}$ denote the flow rate of the $2^{nd}$-order identified and PCA-based reduced order LPV models, respectively.

The mean absolute errors in Table 4 are for $5~\textrm{s}$ starting from $8~\textrm{s}$ and ending at $13~\textrm{s}$. Selecting this time range identifies the transient errors and evaluates the proposed approach accurately. The error analysis shows that the proposed approach provides a response comparable to the experimental measurements. High value of $E_{e,pca}$ for $f=12~\textrm{Hz}$ is due to the poor performance of the model for decrease in flow (see Fig. 11). Error analysis advocates the use of a PCA-based reduced LPV model over the $2^{nd}$-order identified model.

**Table 4:** Error analysis of the models by Eq. (15).

*f*	Increase in flow			Decrease in flow		
	$E_{e}$	$E_{e,2p}$	$E_{e,pca}$	$E_{e}$	$E_{e,2p}$	$E_{e,pca}$
6	0.041	0.21	0.080	0.0034	0.038	0.078
12	0.0057	0.029	0.044	0.16	0.56	0.86
18	0.081	0.19	0.036	0.0079	0.46	0.0071
24	0.18	0.44	0.18	0.0044	0.32	0.0033
30	0.0061	0.28	0.0034	0.0018	0.24	0.0032
36	0.0023	0.26	0.0067	0.0081	0.19	0.0071
42	0.0049	0.16	0.0030	0.0010	0.16	0.00024
48	0.0019	0.13	0.00024	0.0034	0.16	0.0028
54	0.00091	0.12	0.0032	0.0043	0.12	0.0053
60	0.011	0.11	0.013	0.0048	0.021	0.0061

Synthesizing an LPV controller from the proposed model can be challenging because it requires solving $2^M$ Linear Matrix Inequalities (LMIs) which presents high computational complexity. The complexity can be reduced by developing an LPV model with fewer varying parameters. It will be the future work of this research.

## Conclusion

With the proposed an automated data acquisition method, we recorded $\textrm{60}$ input-output data sets by configuring the variable frequency drive and collecting data from a turbine flowmeter. The output responses present a nonlinear behavior of the system for low input frequencies. Moreover, the model identification results in distinct sets of parameters for different inputs. Designing a generalized controller to achieve the desired flow rate requires a universal model that represents the dynamics of the system for the entire input range. Since model order directly impacts the computational burden on control design and its real-time implementation, identification of a low-ordered model is a crucial aspect. Our approach identifies $3^{rd}$-order TFs for distinct input-output data sets, applies PCA-based transformation to obtain $2^{nd}$-order TFs, and constructs an LPV model for the system. This way, we achieve a universal $2^{nd}$-order TF with varying parameters. The variation of the parameters depends on the real-time measurements and change in the dynamic behavior of the system based on the change in the input frequency.

The resulting estimated model can be used to synthesize a gain scheduling or an LPV controller for the system using linear time invariant methods of control design. The mean absolute errors and comparison with the experimental data ensure that the reduced-order LPV model outperforms the identified model of $2^{nd}$-order. Although the proposed method presents computational complexity, it is practical and offers simplicity from an implementation viewpoint.

## Data Availability

The datasets used and/or analysed during the current study are available from the corresponding author on reasonable request.
